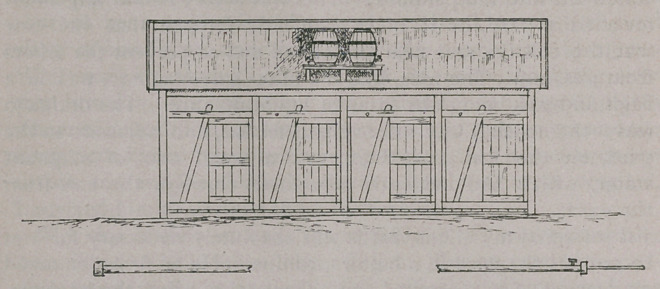# An Outbreak of Epizoötic Abortion in Cattle

**Published:** 1895-08

**Authors:** 

**Affiliations:** Veterinary Surgeon; Warrnambool, Victoria, Australia


### THE JOURNAL
OF
### COMPARATIVE MEDICINE AND VETERINARY ARCHIVES.

VOL. XVI. *AUGUST*, 1895. No. 8.

### AN OUTBREAK OF EPIZOÖTIC ABORTION IN CATTLE.[1]

BY VETERINARY SURGEON DESMOND,
WARRNAMBOOL, VICTORIA, AUSTRALIA.

As much attention is being drawn to the subject of epizoötic abortion, the following results of investigation may be of interest to those studying the subject, and of service to country practitioners:

An outbreak occurred in this district on a large dairy farm, where upward of eighty dairy-cows were being milked, the milk supplying a cheese- and butter-factory. The farmer came to me with the doleful cry, "I am in dreadful trouble. I have lost £100 on my farm during the last few months." He went on to explain that forty cows had slipped their calves; and on further questioning I ascertained that the animals had aborted in all stages of gestation, from a few months to near full term, or, to repeat his own words, "They were from as big as my boot to full size."

I at once came to the conclusion that I would have to treat an outbreak of epizoötic abortion, and a subsequent inspection of the cattle and farmyard confirmed me in this beyond a doubt.

I first proceeded to examine my client's stock and the surroundings of the farm. It was about an hour after the morn-

[1] Read at the June meeting of the Missouri Valley Veterinary Medical Association, by Dr. R. H. Harrison, D.V.S.

ing's milking when I arrived, and the cows which had just been milked were to be seen in an adjacent paddock, where green rye-grass was supplied to them morning and evening, this being distributed over the ground in small heaps. The cattle were of the class generally to be seen on dairy-farms in the district, and were of various ages and of all sizes. The cows that had aborted were easily picked out of the herd, as they showed want of condition, and their hair was rough and lustreless. While walking through the paddock in which the cattle were then grazing, my attention was attracted to several places where the earth had evidently been recently thrown up, and the farmer confirmed my suspicions by saying that it had been the custom to bury the aborted fœtuses where they were expelled. I advised him to destroy them by burning.

The dry cattle were next inspected, and the cows especially were found to be in splendid condition. But on entering a paddock in which the young cattle (all heifers) were grazing, I was surprised to see a very poor bull. On closer examination I found him to be in wretched condition, and the brute had scarcely sufficient strength to move about. His hair was very rough, while the tuft of hair at the prepuce was matted and had a dark appearance, which made me suspicious that he had been the means of assisting to spread the disease. The owner informed me that he had paid £3 for this bull, and had had him two years. I instructed him to destroy the animal at once. "That is the only one I have," protested my client, and in reply to my question whether he thought it right to have only one bull to one hundred cows, he said he considered it quite enough, though he had often seen six or seven cows in season around the bull while it was lying down. I informed him that thirty cows to one bull were considered by competent authorities to be a sufficient number.

An inspection of the milking-yard disclosed that it was in a deplorable state. It was more than two feet deep with liquid manure, and the corners, which were slightly lower than other portions of the yard, were covered with a green scum. The reason assigned for allowing the filth to accumulate in this manner was that the manure was required, and it was only when the yard became too boggy that all the stuff was removed, and a layer of ferns spread over it. The yard was in a very bad situation, and the arrangements were on a primitive plan. It was three chains from the dwelling-house, and was only threequarters of a chain square, being altogether too small for the number of cattle (eighty) milked there twice a day. The yard sloped toward the bales, which were built under an old shed on one side, and so arranged that each cow after being milked had to go through a small gate at the head of the bale into a race which led into the paddock. This was a very bad arrangement for a dairy-yard, as it must frequently happen that one cow would block the race near the outlet, and so prevent the others from passing. The race, like the other surroundings, was in a frightful state, being one mass of liquid manure. The drainage (save the mark!) was also very defective, and adjacent to the yard, on the low side, there was a small pool of stagnant water, which had evidently percolated through the soil from the yard.

I informed my client that it was absolutely necessary for him to remove the yard to a better position. He at first demurred on account of the expense, but consented to effect the improvement when I told him I desired to be paid by results. I suggested that he should request his landlord to pay a portion of the cost of removing the yard, but he seemed to think that there was very little prospect of such an appeal being successful. This landlord was a sheep-breeder, and if he could not get a tenant for the farm for dairying, he could stock it with sheep. [If he did so he would probably soon learn to his cost that an outbreak of this kind is as serious in sheep-raising as it is in dairy-farming.] I selected a more suitable site on which the new yard was to be erected, and was fortunate in finding at a short distance a stony formation which would afford natural drainage. In accordance with my suggestion the shed to contain the bales was built of new timber, as the old timber could not well be disinfected. The floor of the shed was boarded with strong planks with tar joints, and this sloped into a plank drain, also having tar joints, which carried the excreta and disinfecting solution clear of the yard. The gates by which the cattle were let out after being milked were made so as to open directly into the paddock.

I shall now proceed to describe the treatment I determined to put into force: an antiseptic method of treating the outbreak on a plan which I believe is original. For this purpose a high stage was erected in the yard at the rear of the bales, and on this stage two casks, each of thirty gallons capacity, were mounted sufficiently high to allow the solution to gravitate with the force required to irrigate the genital organs of the cows—one cask containing a solution for this purpose, while the other was to sluice out the bales and drain. A long pipe with suitable connections was attached to the cask, and by this means the genital organs of the cows were irrigated with a medicated solution. After the milking the same pipe was connected with the other cask, which contained a disinfecting solution, and by this means the shed and bales were disinfected. The following were the ingredients in the solution used for irrigating the genital organs of the cows:

| | |
|---|---|
| Hydr. perch. | 1¼ ounces. |
| Sodii chlorid. | 3 pounds. |
| Rain-water | 30 gallons. |

To disinfect the shed and bales I decided to use the following solutions alternately, and placed in cask No. 2:

| | |
|---|---|
| Hydr. perch. | 5 ounces. |
| Rain-water | 30 gallons. |

and

| | |
|---|---|
| Ferri sulph. comm. | 3 pounds. |
| Rain-water | 30 gallons. |

I gave the following directions to my client: To watch all cows in calf, and immediately there were signs of parturition or abortion to have them removed at once to a small paddock. If abortion took place, the fœtus and placenta were to be destroyed by fire. If the placenta did not come away naturally in ten to twelve hours, to remove it by mechanical means, and then irrigate the genital organs with about two gallons of solution from the No. 1 cask. This treatment to be adopted also with cows that had calved, so as to be on the safe side, and was to be done after all the other cows were milked and turned into pasture. I would have had separate bales and yards for newly-calved cows and also for all new cases of abortion, but I adopted the above plan for the sake of economy.

After the new yards were built and the treatment had been followed for about five days I again visited the farm, and arrived before the morning milking had commenced. An inspection revealed a very altered state of affairs, which tended to show that the farmer had realized the importance of adopting the measures recommended for stamping out the disease. The bales and yard and surroundings were perfectly clean, and there was not the slightest odor from them. I inquired how the treatment had affected the cows, and the reply was, "The amount of matter that came away from the cows was surprising; it was yellow, and in some cases lumps like honeycomb also came away." "What length of time has elapsed since abortion took place in the cows from which there was so much discharge?" was my next question, and to this my client replied, "At all periods, from a few weeks to many months, but the cows the most discharge came from were those that I could get two feet of pipe into the genital organs."

While on this visit I found all the cows in *oestrum*, which I was informed came on about the third day the solution was used. The farmer also mentioned that in the first and second days he could only pass a foot of the pipe into the genital organs, but on the third day he could pass two feet, and was surprised to see such a mass of mucus come away. The fact that all the cows were coming in season was rather an advantage, as the os uteri would be dilated and the uterus could be irrigated.

I have continued the treatment described above, and, so far, the results are highly satisfactory.

One method of spreading the disease came under my notice on the occasion of my last visit. There was a bull near the milking-yard, which, the farmer informed me, belonged to his neighbor, and it had jumped a three-railed fence to get among his cows. I advised him to inform his neighbor of the danger of the disease being carried to his cattle by the agency of the bull, and I believe the animal was at once removed.

In the course of conversation with some professional friends, I was asked, "What would you do if the owner of such a filthy farm as you have described refused to make the improvements?" My reply was that the farmer in this case kept the premises in that state from a mistaken idea of economy rather than from wilful neglect; but in the event of an owner refusing to comply with my instructions I would report the matter to the board of health, and if this did not bring about the desired result then I would inform his landlord and also the persons to whom he supplied the produce. In adopting such measures I would not be exceeding my duties as a veterinarian. If a fatal disease were to break out in that man's family, and the board of health found the premises to be in such an unsanitary condition, then I would be censured, and deservedly so, if it were found that I had previously examined the place and not furnished a report to the authorities.

The outbreak occurred in 1890, so I have had ample time to note the effects of my treatment. It is hardly needful to say that I have waited anxiously for the results, and that I have much pleasure in reporting that they were most gratifying to the owner of the dairy-farm and to myself. I have kept continuously in touch with the owner, and requested him to call on me when anything of note occurred in the course of the treatment. To sum up, the treatment had the desired effect. I think I can say this truthfully from a review of the circumstances. If the irrigation had not been resorted to, how would all the discharge that came away after the irrigation was used have been got rid of? Only four cows aborted at next calving, and the owner informs me that he could not irrigate these four satisfactorily, as he could not get all the pipe into the genital organ.

Since the outbreak was stamped out, Dr. Ross has procured a complete bacteriological laboratory, and has accepted me as his collaborator, and if I had to treat such an outbreak again I would make a bacteriological investigation with a view to isolating the micro-organism concerned in the production of the disease.

## Figures and Tables

**Figure f1:**